# Effects of epigenetic pathway inhibitors on corticotroph tumour AtT20 cells

**DOI:** 10.1530/ERC-19-0448

**Published:** 2020-01-13

**Authors:** K E Lines, P Filippakopoulos, M Stevenson, S Müller, H E Lockstone, B Wright, S Knapp, D Buck, C Bountra, R V Thakker

**Affiliations:** 1 OCDEM, Radcliffe Department of Medicine, University of Oxford, Churchill Hospital, Oxford, UK; 2 Structural Genomics Consortium, University of Oxford, Oxford, UK; 3 Structural Genomics Consortium, Buchmann Institute for Life Sciences, Goethe-University Frankfurt, Frankfurt, Germany; 4 Oxford Genomics Centre, Wellcome Trust Centre for Human Genetics, University of Oxford, Oxford, UK; 5 Institute of Pharmaceutical Chemistry, Goethe-University Frankfurt, Frankfurt, Germany

**Keywords:** bromo and extra terminal domain, histone code reader, pituitary, Cushing’s disease, neuroendocrine

## Abstract

Medical treatments for corticotrophinomas are limited, and we therefore investigated the effects of epigenetic modulators, a new class of anti-tumour drugs, on the murine adrenocorticotropic hormone (ACTH)-secreting corticotrophinoma cell line AtT20. We found that AtT20 cells express members of the bromo and extra-terminal (BET) protein family, which bind acetylated histones, and therefore, studied the anti-proliferative and pro-apoptotic effects of two BET inhibitors, referred to as (+)-JQ1 (JQ1) and PFI-1, using CellTiter Blue and Caspase Glo assays, respectively. JQ1 and PFI-1 significantly decreased proliferation by 95% (*P* < 0.0005) and 43% (*P* < 0.0005), respectively, but only JQ1 significantly increased apoptosis by >50-fold (*P* < 0.0005), when compared to untreated control cells. The anti-proliferative effects of JQ1 and PFI-1 remained for 96 h after removal of the respective compound. JQ1, but not PFI-1, affected the cell cycle, as assessed by propidium iodide staining and flow cytometry, and resulted in a higher number of AtT20 cells in the sub G1 phase. RNA-sequence analysis, which was confirmed by qRT-PCR and Western blot analyses, revealed that JQ1 treatment significantly altered expression of genes involved in apoptosis, such as NFκB, and the somatostatin receptor 2 (SSTR2) anti-proliferative signalling pathway, including SSTR2. JQ1 treatment also significantly reduced transcription and protein expression of the ACTH precursor pro-opiomelanocortin (POMC) and ACTH secretion by AtT20 cells. Thus, JQ1 treatment has anti-proliferative and pro-apoptotic effects on AtT20 cells and reduces ACTH secretion, thereby indicating that BET inhibition may provide a novel approach for treatment of corticotrophinomas.

## Introduction

Corticotrophinomas represent >10% of all surgically removed pituitary adenomas, which are the most commonly encountered intracranial neoplasms that are identified in >25% of unselected autopsies and approximately 20% of the population undergoing intracranial imaging (Daly* et al.* 2009). Corticotrophinomas are associated with hypersecretion of adrenocorticotropic hormone (ACTH), which leads to excessive production of glucocorticoids by the adrenal cortex, and the resulting hypercortisolemia causes Cushing’s disease, whose clinical features include obesity, redistribution of adipose tissue, muscle atrophy with preclinical myopathy, diabetes mellitus, hypertension, osteoporosis, subfertility, skin thinning, depression, psychosis and increased susceptibility to infection (Daly* et al.* 2009, Ntali* et al.* 2013, Yates* et al.* 2015). Corticotrophinomas are therefore a cause of ACTH-dependent Cushing’s disease, which is also referred to as pituitary-dependent Cushing’s syndrome (Ntali* et al.* 2013). Corticotrophinomas, which are neuroendocrine tumours (NETs) of pituitary, are usually microadenomas (i.e. <10 mm in diameter) and often are too small to be detected by radiological imaging (e.g. MRI or computerised tomography (CT) scans) or identifiable at surgery (Cuevas-Ramos* et al.* 2016).

The treatment of choice for corticotrophinomas is transsphenoidal resection, which results in remission rates of 70–90% for microadenomas (Cuevas-Ramos* et al.* 2016). However, mortality rates of 1–2% are reported to be associated with transsphenoidal resection, and long-term (10 years) recurrence rates of ~20% following transsphenoidal resection indicate that a long-term cure is achieved in only ~60–80% of adults with corticotrophinomas (Daly* et al.* 2009, Cuevas-Ramos* et al.* 2016). Pharmacological treatments are available for patients for whom transsphenoidal surgery has not been successful in removing the corticotrophinomas and these include inhibitors of steroidogenesis (e.g. metyraprone, ketoconazole, mitotane, etomidate and osilodrostat); glucocorticoid antagonists (e.g. mifepristone); dopamine agonists such as cabergoline; and somatostain analogues such as pasireotide (Cuevas-Ramos* et al.* 2016). However, these current medical treatments for corticotrophinomas have limited efficacy, and thus, there is a clinically unmet need for improved pharmacological treatments for corticotrophinomas, especially for those occurring in patients who have contraindications for surgery or have had unsuccessful surgery.

Epigenetic-targeting compounds are a new class of anti-tumour drugs, and one such family of small molecule bromo and extra-terminal domain (BET) inhibitors, which target the bromodomains (BRDs) of the protein family members BRD2, BRD3, BRD4 and BRDT that bind acetylated residues on histones that regulate gene expression, and particularly those of tissue-specific genes (Filippakopoulos* et al.* 2010), have been shown in preclinical *in vitro* and *in vivo* studies to have efficacy in a number of tumour types including pancreatic neuroendocrine tumours, glioma, nuclear protein in testis (NUT)-midline carcinoma, leukaemias and renal cell carcinoma (Beesley* et al.* 2014, Coude* et al.* 2015, Ishida* et al.* 2017*a*,*b*, Leal* et al.* 2017, Lines* et al.* 2017, Wu* et al.* 2017). Moreover, in order to determine if BET inhibitors may also represent an effective novel therapy for corticotrophinomas in reducing proliferation and increasing apoptosis of these pituitary cells, we first investigated the mouse corticotroph tumour cell line AtT20 for expression of the BET protein family members and then the effects of the BET inhibitors JQ1 and PFI-1 on proliferation, apoptosis and ACTH secretion by these pituitary cells.

## Materials and methods

### Cell lines, *in vitro* assays and compounds

AtT20 murine pituitary corticotroph tumour cells were purchased from the American Type Culture Collection (ATCC) (CCL-89); murine cells that were used as a human corticotroph tumour cell line is not currently available. AtT20 cells are small, round, adherent cells that have a doubling time of approximately 1–2 days and were originally isolated from a LAF1 mouse pituitary tumour (Buonassisi* et al.* 1962). Cells were cultured in DMEM media, supplemented with 10% foetal calf serum (FCS) (Sigma-Aldrich), maintained at 37°C, 5% (vol/vol) CO_2_ and tested for mycoplasma using the MycoAlert kit (Lonza). PFI-1, (+)-JQ1 (henceforth JQ1) and its inactive control compound ((-)-JQ1, henceforth JQ1-) were suspended and diluted in dimethyl sulfoxide (DMSO, Sigma-Aldrich), as previously described (Lines* et al.* 2017). Both compounds were obtained from the Structural Genomics Consortium (SGC, University of Oxford), and further details on the structure and specificity for each compound are available at https://www.thesgc.org/chemical-probes. Octreotide (Sigma-Aldrich) was suspended and diluted in distilled water. Untreated and vehicle (DMSO-only)-treated AtT20 cells were used as controls. For all studies, cells underwent trypsin treatment, before the cell number was determined by trypan blue staining and counting using a haemocytometer. Proliferation, apoptosis and senescence assays were performed in 96-well plates with 5000 cells seeded per well, 24 h before drug treatment. For cell cycle analysis, 50,000 cells were seeded per well in 24-well plates, 24 h before drug treatment. Cell viability, as an indication of cell proliferation, was assessed using the CellTiter Blue Cell Viability assay (Promega), whereby 20 µL of CellTiter Blue reagent was added per well, incubated for 2 h at 37°C, 5% (vol/vol) CO_2_ and the fluorescent outputs read on a CytoFluor microplate reader (PerSeptive Biosystems, MA, USA) at 530 nm excitation and 580 nm emission (Eachkoti* et al.* 2014). Cell death by apoptosis was evaluated using the CaspaseGlo 3/7 assay (Promega), whereby 75 µL of CaspaseGlo reagent was added per well, incubated for 1 h at room temperature and the luminescent outputs read on a Turner Biosystems luminometer. Cellular senescence was determined using the Cellular Senescence Assay (SA-β-gal staining) kit (Cell Biolabs, CA, USA) (Lee* et al.* 2015), 96 h after drug treatment. Cells were visualised using an Eclipse E400 microscope (Nikon), and images were captured using a DXM1200C digital camera and NIS-Elements BR 2.30 software (both Nikon). The percentage of positively stained cells were counted, per total cells, from *n* = 20 10× magnification fields. Cell cycle progression was determined using proidium iodide (PI) (Sigma-Aldrich) staining, 48 h after drug treatment. Cells were fixed in 100% ethanol, washed with PBS and DNA stained by incubation with PI solution (10 µg/mL PI, 200 µg/mL RNase, 0.1% Triton-X, 150 mM sodium chloride; diluted in PBS) for 30 min at room temperature. Intensity of PI staining was detected on a FACS calibur flow cytometer (Becton Dickinson, NJ, USA) using Cellquest pro software. Peaks of fluorescence, representing DNA content, were used to evaluate the percentage of cells at each cell cycle phase, using FloJo software, as previously described (Lines* et al.* 2017).

### Quantitative real-time PCR (qRT-PCR)

Total RNA from AtT20 cells was extracted using the miRVana kit (Ambion, Thermo Fisher Scientific) and 1 µg used to generate cDNA using the Quantitect RT kit (Qiagen), as described (Lines* et al.* 2017). Gene-specific Quantitect primers (Qiagen) were used for qRT-PCR reactions, which utilised the Quantitect SYBR green kit (Qiagen), on a RotorGene 5, as described (Lines* et al.* 2017). Each test sample was normalized to the geometric mean of reference genes glyceraldehyde 3-phosphate dehydrogenase (*Gapdh*) and calnexin (*Canx*). The relative expression of target cDNA in all qRT-PCR studies was determined using published methods (Pfaffl 2001).

### RNA-sequence (RNA-Seq) analysis

Confluent AtT20 cells were treated with 1 µM JQ1- or JQ1 for 48 h before RNA extraction using the RNeasy kit (Qiagen). RNA sequencing was performed on three independent experimental replicates for each cell line and treatment. Following polyA-enrichment and library preparation, 50 bp paired-end sequencing was performed (Illumina 2500 machine, rapid mode, 2 lanes). Reads were aligned to the mouse reference genome (GRCm37) using TopHat2 (Kim* et al.* 2013) and duplicate reads were removed (Picard Tools MarkDuplicates). Approximately 22–30 million high-quality reads per sample were mapped uniquely to Ensembl-annotated genes; gene counts were summarised using HTSeq (Anders* et al.* 2015) and filtered to exclude genes with fewer than ten reads on average per sample. The filtered set of genes were further analysed in the R statistical software (https://www.r-project.org/) to characterise data quality and sample clustering patterns. Data normalisation and differential expression analysis, comparing JQ1 and JQ1- treatment conditions, were performed using the edgeR package (Robinson* et al.* 2010). Genes with adjusted *P* value <0.05 and showing a fold change >2 in either direction were considered significant. Identification of altered cellular pathways was undertaken using QIAGEN Ingenuity® Pathway Analysis (IPA®, QIAGEN, www.qiagen.com/ingenuity). Gene-set enrichment analysis (GSEA) against MSigDB (software.broadinstitute.org/gsea/msigdb) gene-sets (v.6.2) was performed using the *fgsea* package in R/Bioconductor (http://bioconductor.org/packages/fgsea/) after mapping mouse EntrezIDs to human using the *biomaRt* package (Durinck* et al.* 2009, Sergushichev 2016).

### Western blot analysis

AtT20 cells were lysed in NP40 lysis buffer: 250 mM NaCl, Tris 50 mM (pH 8.0), 5 mM EDTA, 0.5% NP-40 (v/v) and 2× Protease inhibitor tablets (Roche), as described (Lines* et al.* 2017). Lysates were prepared in 4× Laemmli loading dye (BioRad) boiled at 95°C for 5 min, resolved using 6% or 10% SDS-PAGE gel electrophoresis, transferred to polyvinylidene difluoride membrane and probed with primary antibodies (Brd2 – D89B4, Cell Signalling; Brd3 – 61489, Active motif, La Hulpe, Belgium; Brd4 – PA5-51550, Thermo Fisher Scientific; NFKβ1 (ab32360), cIAP2 (ab23423), Src (ab47405), POMC (ab94446) and SSTR2 (ab134152) all - Abcam; Calnexin – ab2301, Millipore; and *Gapdh* – ab181603, AbCam) and HRP-conjugated secondary antibody (sc-2357, Santa Cruz Biotechnology) prior to visualisation using Pierce ECL Western Blotting substrate (Thermo Fisher Scientific), as described (Lines* et al.* 2017). Calnexin or *Gapdh* protein expression was used as a loading control, and lysates from human bronchopulmonary neuroendocrine cells (H727) were used as a positive control (Lines* et al.* 2017). Densitometry analysis was performed by calculating the number of pixels per band using ImageJ software. Data were represented as the number of pixels of the protein band, relative to the number of pixels of the corresponding *Gapdh* or Calnexin band.

### ACTH enzyme-linked immunosorbant assay (ELISA)

AtT20 cells were cultured in 24-well plates in 1 mL media. Cells were counted, harvested and media collected at 0, 24, 48 and 72 h after JQ1- or JQ1 treatment. Harvested cells were lysed and used for Western blot analysis. An ELISA was performed on the cell media to assess for ACTH secretion. Briefly, media was diluted 1:3 in distilled water and 200 μL added per well of a mouse/rat ACTH ELISA kit (Origene, MD, USA), incubated for 4 h with biotinylated and enzyme-labelled antibodies, before visualising with 3,3′,5,5′-Tetramethylbenzidine (TMB) substrate and reading at 405 nM on a Pherastar FS (BMG LabTech, Ortenberg, Germany). Data were normalised to 0 h ACTH levels and corrected for cell number per well to avoid bias through alterations in proliferation and apoptosis.

### Statistical analysis

Data were analysed using one-way or two-way ANOVA correcting for multiple comparisons or Dunnett’s test for a single control group, as previously described (Walls* et al.* 2012, Gorvin* et al.* 2013). For *in vitro* data, comparisons were relative to vehicle, and for the ACTH ELISA and RNA-Seq data, comparisons were relative to JQ1- treatment. RNA-Seq data were analysed using statistical models implemented in the edgeR package (Robinson* et al.* 2010). RNA-Seq results are reported with adjusted *P* values, after correcting raw *P* values for multiple testing using and Benjamini–Hochberg’s method (Benjamini & Hochberg 1995) to control the false discovery rate (FDR) at 5%.

## Results

### AtT20 cells express members of the BET family, with Brd2 being the most abundant

**Figure 1:**
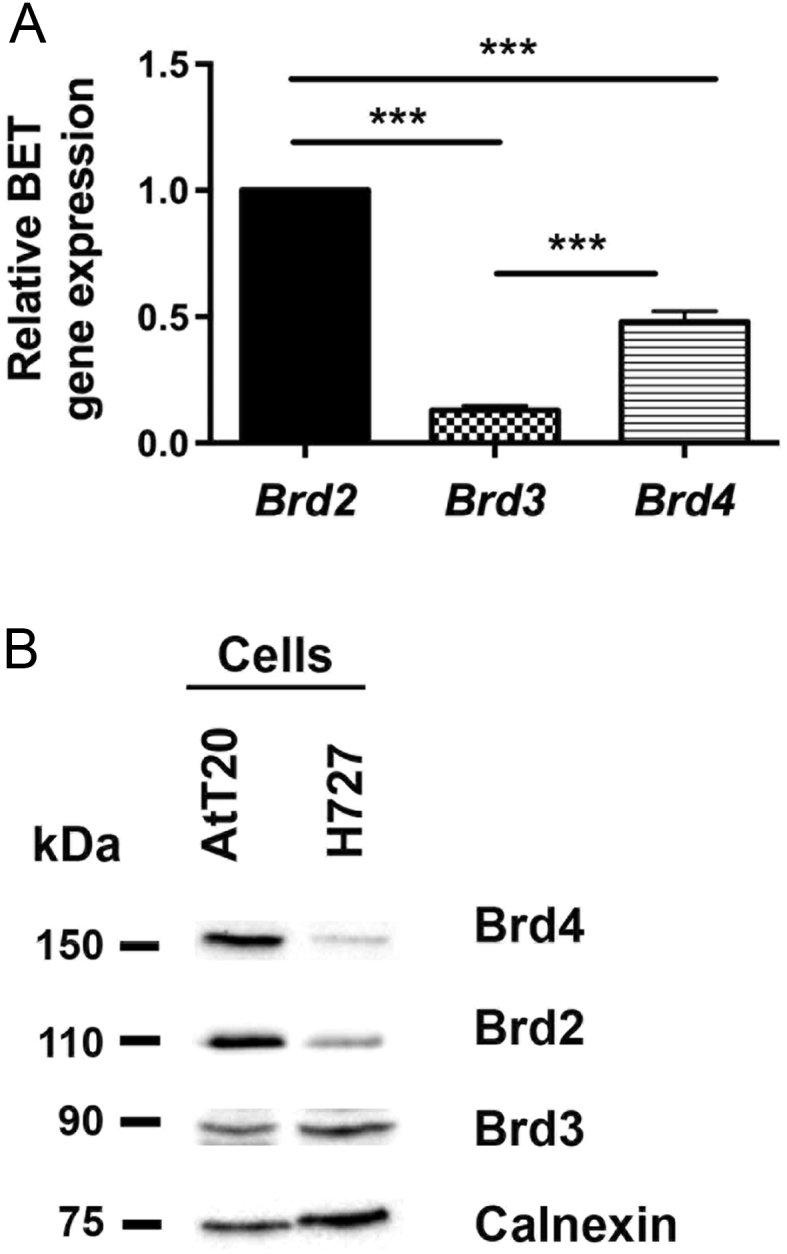
Expression of BET family members in AtT20 cells. The mRNA expression of the BET family members *Brd2*, *Brd3* and *Brd4* was examined in AtT20 cells by qRT-PCR (A). Data from four cell passages are represented relative to *Brd2* expression; ***P < 0.0005. Western blot analysis was used to confirm expression of BRD2, BRD3 and BRD4 proteins (B), with representative data from four cell passages shown; H727 cell lysate was used as a positive control, and calnexin (a housekeeper gene) was used as a loading control.

Evaluation of the expression of the four BET family members *Brd2*, *Brd3*, *Brd4* and *Brdt* in AtT20 cells by quantitative real-time PCR (qRT-PCR) revealed that *Brd2* is the most abundant member with 7.7-fold higher expression than *Brd3* (*P* < 0.0005) and 2.1-fold higher expression than *Brd4* (*P* < 0.0005) (Fig. 1A). *Brd4* was the second most abundant BET family member, with 3.7-fold (*P* < 0.0005) higher expression than *Brd3* (Fig. 1A). Expression of *Brdt*, which is testis specific, was not detected after 40 cycles of the qRT-PCR. Western blot analysis confirmed expression of BRD2, BRD3 and BRD4 proteins in AtT20 cells (Fig. 1B).

### JQ1 and PFI-1 reduce proliferation of AtT20 cells

**Figure 2:**
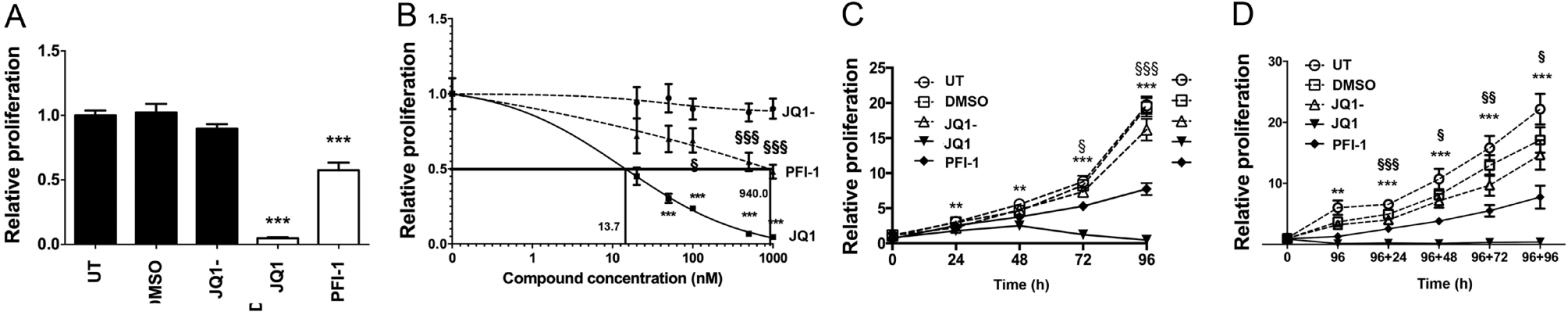
Effects of BET inhibitors JQ1 and PFI-1 on proliferation of AtT20 cells. JQ1 and PFI-1 significantly reduced proliferation of AtT20 cells, when compared to untreated (UT) cells, DMSO-only treated cells and JQ1-treated cells. Proliferation was assessed using CellTiter Blue assays after 96 h treatment with 1 µM of each compound. Control treatments are represented by black bars and test compound treatment by white bars (A). Dose escalation studies using 20 nM, 50 nM, 100 nM, 500 nM and 1 µM JQ1 or PFI-1 and their effects on proliferation of AtT20 cells after 96 h treatment. The EC_50_ value is indicated by a solid line. Responses to JQ1 (filled squares and solid line), JQ1- (filled circles and broken line) and PFI-1 (filled triangles and broken line) are shown (B). The efficacy of the BET inhibitors JQ1 and PFI-1 was further examined by time course analysis of cell proliferation after a single treatment with 1 µM JQ1 or PFI-1. Cells were treated with 1 µM JQ1-, JQ1 or PFI-1 and proliferation measured every 24 h and up to 96 h following treatment. Control treatments are represented by dashed lines and drug treatments by solid lines (C). After 96 h treatment with 1 µM JQ1-, JQ1 or PFI-1, compounds were removed (indicated by the arrow) and AtT20 cells culture in standard culture media. Proliferation was measured every 24 h for the following 96 h (D). For all experiments, significance is relative to DMSO vehicle only treatment with (A) ***P < 0.0005 and in (B, C and D) PFI-1 statistics represented by the symbol § and JQ1 statistics by *; ^§^/*P < 0.05, ^§§^/**P < 0.005 and ^§§§^/***P < 0.0005. For all experiments untreated (UT), vehicle only (DMSO) and JQ1- were used as negative controls, and experiments were performed in *n* = 4 biological replicates, with four technical replicates per experiment.

The effect of JQ1 and PFI-1 on proliferation of AtT20 cells was evaluated after 96 h treatment with 1 µM of each compound and compared to proliferation after treatment with the negative control JQ1-, DMSO (vehicle only) and untreated cells. One µM was selected for both compounds as this dose had previously been demonstrated to have efficacy in cell models, without eliciting non-specific effects (summary of drug properties, toxicity and efficacy available from https://www.thesgc.org/chemical-probes). JQ1 and PFI-1 significantly reduced proliferation by 95% (*P* < 0.0005) and 43% (*P* < 0.0005), respectively (Fig. 2A). Further investigation of proliferation using dose escalation (concentrations of 20 nM, 50 nM, 100 nM, 500 nM and 1 µM) studies revealed JQ1 to significantly reduce proliferation of AtT20 cells with a half maximal effective concentration (EC_50_) value of 13.7 nM, whereas the EC_50_ value for PFI-1 was much higher at 940 nM (Fig. 2B). For all subsequent experiments 1 µM of JQ1 and PFI-1 was used, as the higher dose would ensure maximal cellular responses for both compounds, and also at this dose the negative control compound (JQ1-) did not significantly alter cell proliferation. In addition, JQ1 significantly reduced AtT20 cell proliferation as early as 24 h after treatment (by 39%, *P* < 0.005), whereas PFI-1 only significantly reduced proliferation (by 36%, *P* < 0.05) 72 h after treatment, when compared to control treated cells (Fig. 2C). The effects of JQ1 were maintained following removal of the compound from the culture media, such that 96 h after removal of JQ1 from the culture media, the AtT20 cells had not resumed proliferation, whereas the proliferation of cells treated with PFI-1 had increased by 7.7-fold and 20.2-fold for untreated cells (Fig. 2D).

### JQ1, but not PFI-1, increases apoptosis of AtT20 cells

**Figure 3:**
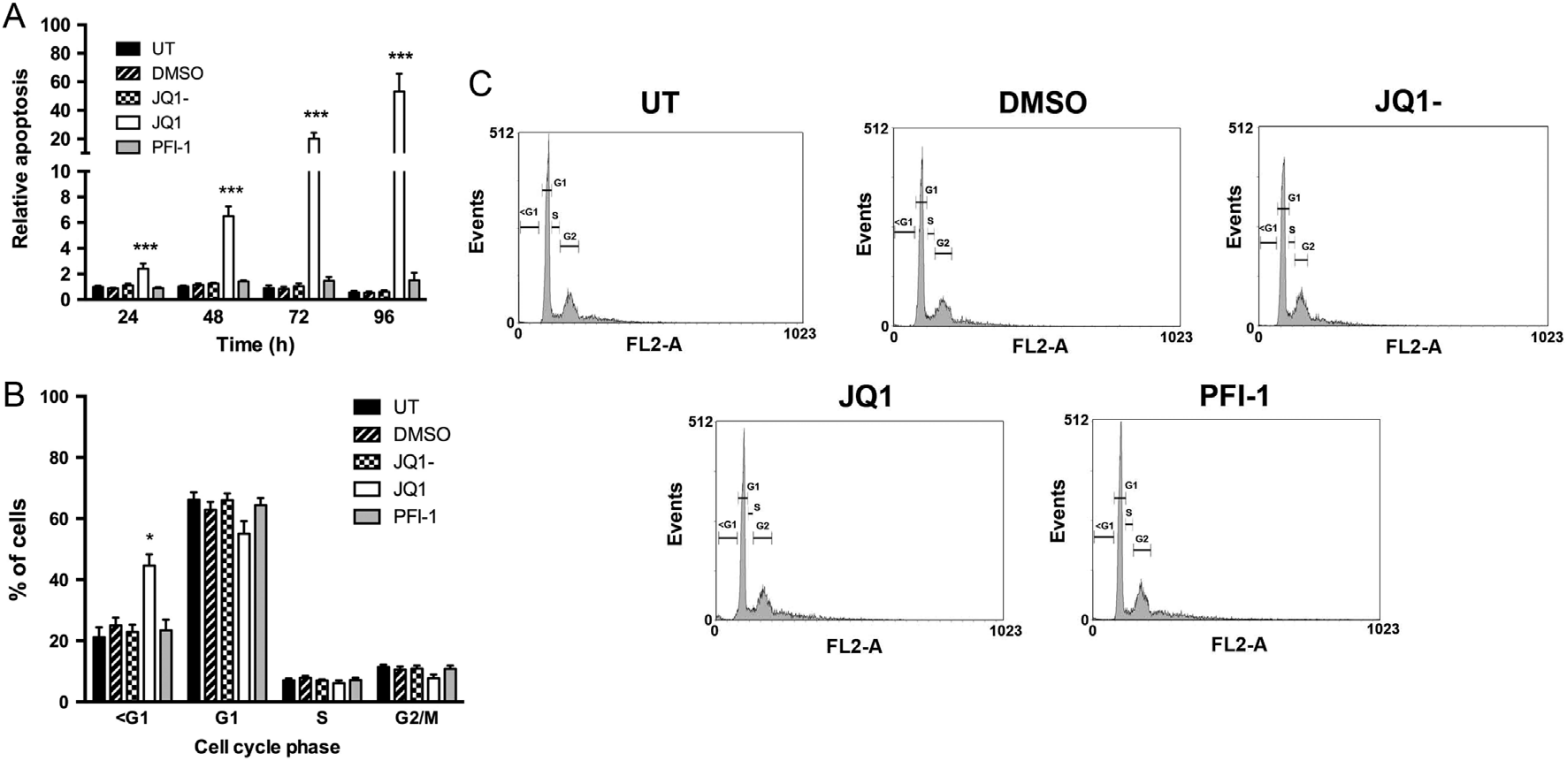
Effect of BET inhibition on apoptosis of AtT20 cells. Apoptosis of AtT20 cells was evaluated every 24 h for 96 h after a single treatment with 1 µM JQ1 or PFI-1. Apoptosis was measured by detection of caspase 3/7 activity (A). Cell cycle profiles were analysed after 48 h compound treatment using propidium iodide staining and flow cytometry (B). Gating for cell cycle phases is indicated on the histograms; FL2-A is the level of fluorescence resulting from the propidium iodide staining (C). UT is represented by black bars, DMSO represented by bars with diagonal lines, JQ1-represented by chequered bars, JQ1 by white bars and PFI-1 treatment by grey bars. For all experiments untreated (UT), vehicle only (DMSO) and JQ1- were used as negative controls, and experiments were performed in *n* = 4 biological replicates, with four technical replicates per experiment. Statistical significance is relative to DMSO vehicle only treatment with *P < 0.05 and ***P < 0.0005.

To assess the ability of JQ1 and PFI-1 to induce apoptosis, the cells were treated with compounds and analysed by Caspase Glo and cell cycle analysis. JQ1 treatment, compared to vehicle control treatment, significantly increased apoptosis, assessed using the Caspase Glo assay, of AtT20 cell lines by 2.4-fold (*P* < 0.0005) 24 h after treatment and up to 53.2-fold (*P* < 0.0005) 96 h after treatment (Fig. 3A). In contrast, PFI-1 had no significant effect on the rate of apoptosis at the concentration used (Fig. 3A). Furthermore, cell cycle analysis using flow cytometry and propidium iodide staining revealed significantly more (by 2-fold, *P* < 0.05) cells in the sub G1 phase of the cell cycle, indicative of apoptosis, when treated with JQ1, when compared to control treated cells (Fig. 3B and C), whereas PFI-1 treatment had no effect on the cell cycle phase of AtT20 cells (Fig. 3B and C). Neither JQ1 nor PFI-1 treatment significantly altered cellular senescence of AtT20 cells, when compared to control treated cells assessed by β-galactosidase staining (Supplementary Fig. 1, see section on supplementary materials given at the end of this article). These results indicate that JQ1 reduces AtT20 cell proliferation through a major pro-apoptotic effect, rather than arresting cells in the G1-G2/M phases of the cell cycle.

### Expression of genes in proliferation- and apoptosis-associated pathways are significantly altered in AtT20 cells after JQ1 treatment

**Figure 4:**
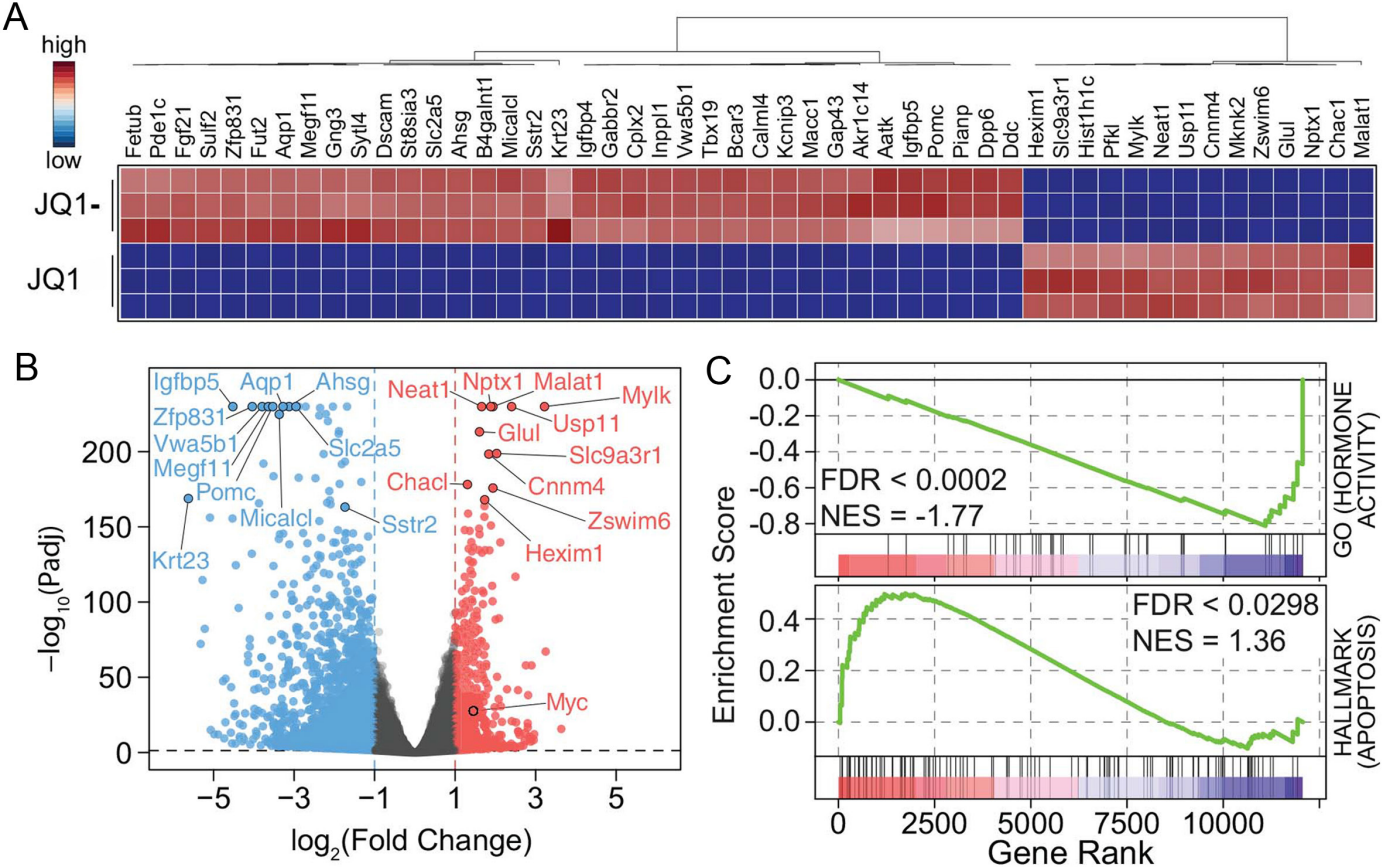
Transcriptional dysregulation in JQ1-treated AtT20 cells. Heatmap of the top 50 significantly (*P* < 0.05) up- and downregulated genes following 48 h treatment with JQ1, including *Fetub*, *Pde1c*, *Fgf21*, *Nptx1*, *Chac1* and *Malat1*. Data shown are fragments per kilobase of exon model per million reads mapped (FPKM) values from RNA-seq experiments, column normalized as indicated in the inset (A). Volcano plot of log2 fold changes of all genes indicates that there were a higher number of significantly downregulated than upregulated genes. The top ten up- or downregulated (lowest *P* values) are highlighted in red and blue respectively. Myc, typically downregulated following BET targeting with JQ1 is also highlighted in the plot (B). GSEA identified significant (*P* < 0.05) enrichment of the GO_HORMONE_ACTIVITY (top) and HALLMARK_APOPTOSIS (bottom) gene-sets from MSigDB. While the hormone activity signature was found to be down-regulated, the apoptosis signature was found to be upregulated. The plots show the running sum for the molecular signature database gene set within the AtT20/JQ1 data including the maximum enrichment score and the leading edge subset of enriched genes (C).

RNA-Seq analysis was performed to identify the genes that may be involved in the decreased proliferation (Fig. 2) and increased apoptosis (Fig. 3) of AtT20 cells after JQ1 treatment. A total of 2365 genes were found to be dysregulated (at 5% FDR and with greater than 2-fold change of expression level), and this comprised 751 genes that were significantly upregulated and 1614 genes that were significantly downregulated in JQ1 treated, compared to JQ1- control treated AtT20 cells. The most significantly highly up- and downregulated genes (Fig. 4A and B) included metastasis associated lung adenocarcinoma transcript 1 (*Malat1*), ChaC glutathione specific gamma-glutamylcyclotransferase 1 (*Chac1*), neuronal prntraxin 1 (*Nptx1*), fetuin B (*Fetub*), phosphodiesterase 1C (*Pde1c*) and fibroblast growth factor 21 (*Fgf21*). In addition, *Myc*, which is a widely reported anti-tumorigenic target of BRD4, was observed in the RNA-Seq data to be upregulated by 2.8-fold in JQ1-treated cells, when compared to JQ1-treated AtT20 cells (Fig. 4B), and this was confirmed by qRT-PCR (Supplementary Fig. 2). Furthermore 68% of genes were observed to be downregulated, while only 32% of genes were observed to be upregulated (Fig. 4B). However, the most highly dysregulated genes were not associated with specific anti-tumorigenic pathways, and GSEA was therefore performed. This indicated that there was a significant enrichment of genes associated with a reduction in hormone activity (FDR < 0.0002) and a significant enrichment of genes associated with increased hallmarks of apoptosis (FDR < 0.0298) (Fig. 4C). Therefore, we focussed on the downregulated genes associated with proliferation, apoptosis and hormone activity in all further analysis.

**Figure 5:**
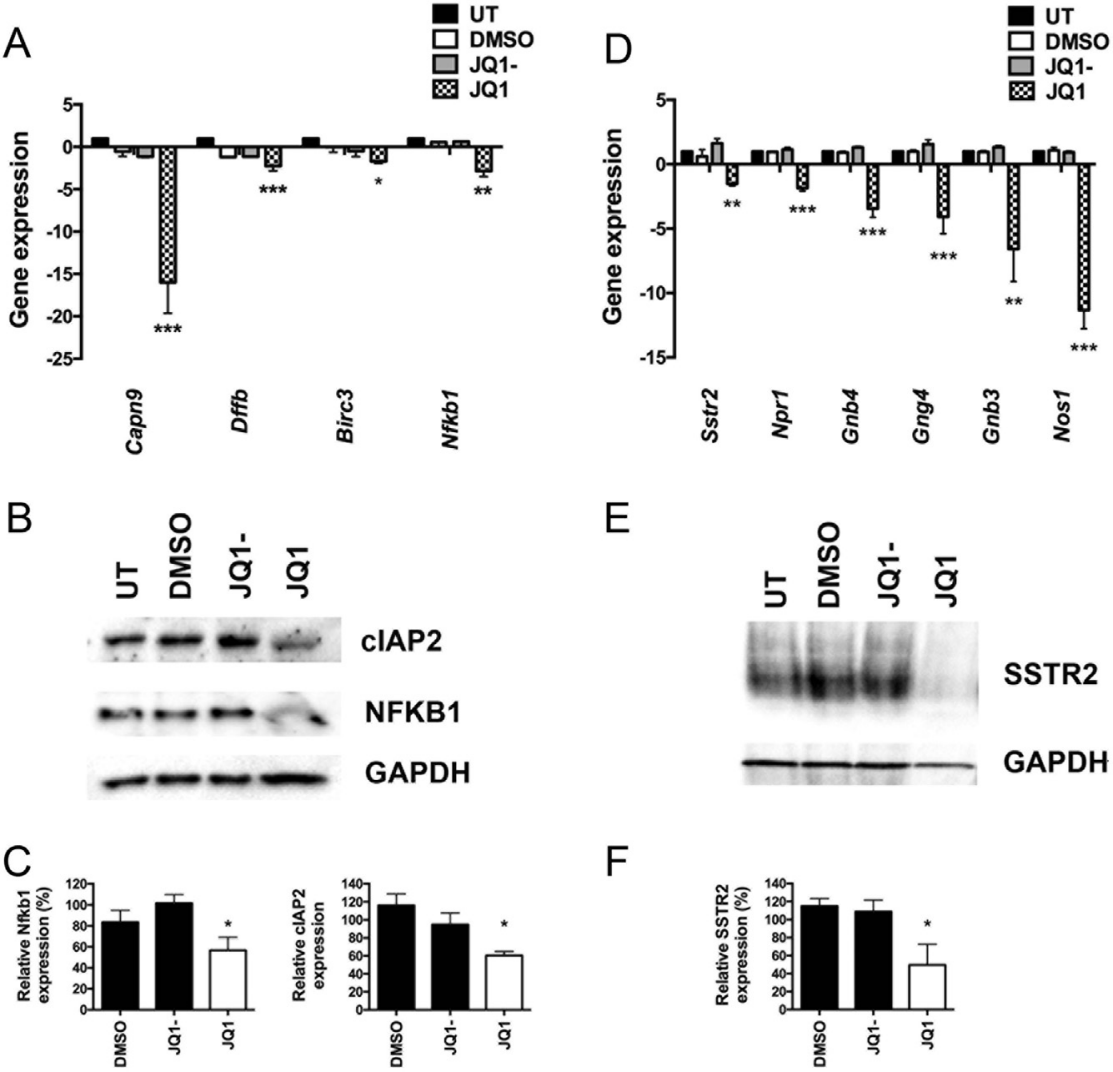
Validation of RNA-Seq data from AtT20 cells treated with JQ1- or JQ1. Transcription of the apoptosis associated genes *Capn9*, *Dffb*, *Birc3* and *Nfkb1* was examined using qRT-PCR, after 48 h JQ1 treatment (A). Expression of cIAP2 (encoded by *Birc3*) and Nfkb1 after JQ1 treatment was confirmed by Western blot analysis (B) and was significantly decreased compared to control treated cells, as indicated by densitometry analysis (C). Transcription of the SSTR2 proliferation pathway associated genes *Sstr2*, *Npr1*, *Gnb4*, *Gng4*, *Gnb3* and *Nos1* was examined using qRT-PCR, after JQ1 treatment (D). The expression of SSTR2 after JQ1 treatment was confirmed using Western blot analysis (E) and was significantly downregulated, compared to control treated cells, as indicated by densitometry analysis (F). For all experiments untreated (UT), vehicle only (DMSO) and JQ1- were used as negative controls, and Gapdh (a housekeeper gene) was used as a loading control. All qRT-PCR experiments were performed in *n* = 4 technical and *n* = 4 biological replicates, and Western blots performed in *n* = 5 biological replicates. Statistical significance is relative to DMSO vehicle only treatment with * *P* < 0.05, ** *P* < 0.005 and *** *P* < 0.0005.

To further identify genes specifically associated with apoptosis pathways, IPA was performed on the 2365 dysregulated genes. In total, 12 apoptosis-associated genes were observed (eight upregulated and four downregulated) in JQ1-treated cells, compared to JQ1- control treated cells (Table 1 and Supplementary Fig. 3). The downregulation of these apoptosis-associated genes was validated using qRT-PCR and confirmed that Nuclear Factor Kappa B Subunit 1 (*Nfkb1*) was downregulated by 2.9-fold (*P* < 0.0005); DNA fragmentation factor subunit B (*Dffb*) was downregulated by 2.3-fold (*P* < 0.0005); Baculoviral IAP Repeat Containing 3 (*Birc3*) was downregulated by 1.7-fold (*P* < 0.005) and Calpain 9 (*Capn9*) was downregulated by 16-fold (*P* < 0.0005) (Fig. 5A). The significant downregulation of NFκB1 (1.5-fold, *P* < 0.05) and cIAP1 (encoded by *Birc3,* 1.9-fold, *P* < 0.05) proteins was also confirmed using Western blot and densitometry analyses (Fig. 5B and C).

**Table 1:** Ingenuity pathway analysis of apoptosis-associated genes and somatostatin regulation of proliferation-associated genes shown to be dysregulated in JQ1-treated AtT20 cells, when compared to JQ1-treated AtT20 cells by RNA-sequence analyses.

Gene	Fold change
Apoptosis-associated	
*Capn6*	7.57
*Endog*	3.03
*Rras2*	2.95
*Map3k14*	2.57
*Tnfrsf1b*	2.23
*Nfkbie*	2.03
*Bcl2l11*	2.03
*Sptan1*	2.00
*Nfkb1*^a^	−2.01^a^
*Dffb*^a^	−2.10^a^
*Birc3*^a^	−2.68^a^
*Capn9*^a^	−12.13^a^
Somatostatin 2-associated proliferation	
*Gng3*	100.43
*Rras2*	2.95
*Src*^a^	−2.95^a^
*Gng4*^a^	−3.32^a^
*Sstr2*^a^	−3.34^a^
*Nos1*^a^	−3.53^a^
*Npr1*^a^	−3.56^a^
*Gnb4*^a^	−4.26^a^
*Gnb3*^a^	−5.78^a^
*Gucy2c*^a^	−10.13^a^

^a^Downregulated genes.

GSEA and IPA analyses also revealed that the only proliferation-associated pathway significantly altered after JQ1 treatment was the ‘somatostatin receptor 2 (SSTR2) anti-proliferative pathway’. This is of interest as somatostatin analogues (e.g. octreotide and lanreotide) inhibit proliferation and hormone secretion by pituitary tumours, such as somatotrophinomas, but not corticotrophinomas (Yates* et al.* 2015, Cuevas-Ramos* et al.* 2016, Walls* et al.* 2016). IPA revealed that JQ1 treatment significantly dysregulated ten genes from this SSTR2 pathway (two upregulated and eight downregulated) when compared to JQ1- treated cells (Table 1 and Supplementary Fig. 4). Analysis by qRT-PCR confirmed downregulation of six of the eight genes that comprised *Sstr2* (by -1.5-fold (*P* < 0.005)); guanine nucleotide binding protein (*G protein*), gamma 4 (*Gng4*) (by -4.1-fold (*P* < 0.0005)); Nitric oxide synthase 1 (*Nos1*) (by -11.3-fold (*P* < 0.0005)); Natriuretic peptide receptor 1 (*Npr1*) (by -1.8-fold (*P* < 0.0005)); Guanine nucleotide binding protein (G protein) beta 4 (*Gng4*) (by -3.4-fold (*P* < 0.0005)) and Guanine nucleotide-binding protein (G protein) beta 3 (*Gnb4*) (by -6.6-fold (*P* < 0.005)) (Fig. 5D). The remaining two genes proto-oncogene tyrosine kinase Src (*Src*) and guanylate cyclase 2C (*Gucy2c*) were found to have undetectable expression. The significant downregulation of SSTR2 (2.3-fold, *P* < 0.05) was also confirmed using Western blot (Fig. 5E) and densitometry analyses (Fig. 5F). Octreotide did not significantly alter the proliferation of AtT20 cells, either alone or in combination with JQ1 treatment (Supplementary Fig. 5).

### JQ1 treatment reduces pro-opiomelanocortin (POMC) expression and alters ACTH secretion of AtT20 cells

**Figure 6:**
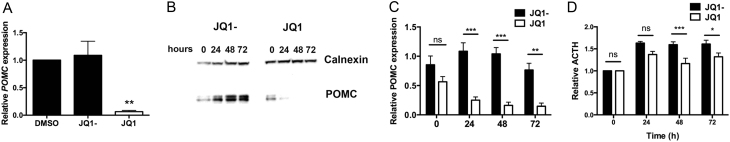
POMC expression and ACTH secretion after JQ1 treatment. QRT-PCR analysis confirmed the significant down-regulation of *Pomc* transcription after 48 h JQ1 treatment (A). Down-regulation of POMC protein expression was also confirmed by Western blot analyses at 24 h, 48 h and 72 h after JQ1 treatment, compared to JQ1- treated cells; POMC is observed as two bands corresponding to pre-pro-POMC and pro-POMC (calnexin was used as a loading control) (B). Densitometry analysis confirmed that POMC expression was significantly down-regulated at 24 h, 48 h and 72 h (C). Concentrations of ACTH in the media from the same cells, which reflected ACTH secretion by these cells, significantly decreased at 48 h and 72 h after treatment with JQ1, when compared to JQ1- treated cells (D). DMSO or JQ1-treated cells were used as negative controls, with qRT-PCR experiments performed in *n* = 4 technical and *n* = 4 biological replicates, and Western blot and ELISAs performed in *n* = 4 biological replicates. Statistical significance is relative to JQ1- treatment with *P < 0.05, **P < 0.005 and ***P < 0.0005.

RNA-Seq analysis indicated that expression of *Pomc*, which encodes the precursor of ACTH, was significantly decreased in JQ1 treated AtT20 cells, compared to JQ1- treated cells, by -11-fold (*P* = 7.31E-320, Fig. 4A and B). This was confirmed using qRT-PCR, which showed that *Pomc* expression was significantly reduced in JQ1 treated AtT20 cells by ~16.7-fold (*P* < 0.005), when compared to vehicle only, and JQ1- treated cells (Fig. 6A). Western blot analysis confirmed that POMC expression was decreased after JQ1 treatment, with significant reduction observed after 24 h (4.4-fold, *P* < 0.0005), 48 h (6.3-fold, *P* < 0.0005) and 72 h (5.1-fold, *P* < 0.005), compared to control JQ1- treated cells (Fig. 6B and C). The reduced POMC expression was associated with significant decreases in the levels of ACTH in cell media at 48 h (1.4-fold, *P* < 0.0005) and 72 h (1.2-fold, *P* < 0.05) after JQ1 treatment, when compared to JQ1- treatment (Fig. 6D).

## Discussion

Our studies have revealed that JQ1 treatment significantly decreased proliferation and increased apoptosis of the ACTH-secreting pituitary tumour cells AtT20. Furthermore, our results demonstrate that the BET protein family regulates the expression of genes in the ‘somatostatin receptor 2 anti-proliferative signalling pathway’ and anti-apoptotic genes, as well as *Pomc*, which encodes the ACTH precursor in these AtT20 cells. Thus, our data indicate that inhibitors of the BET protein family may provide a novel therapeutic strategy for the treatment of ACTH-secreting pituitary NETs.

In our study, we demonstrate that AtT20 cells express the BET family members Brd2, Brd3 and Brd4, with Brd2 being the most abundant, which is similar to data reported for MEN1-associated mouse pancreatic NETs and human pancreatic and bronchial NET cell lines (Lines* et al.* 2017). Thus, the BET family, and specifically Brd2, likely play an important role in NETs, and inhibiting the activity of these proteins may provide a novel therapeutic target. Indeed, our study reveals that the small molecule inhibitors of the BET family, JQ1 and PFI-1, significantly reduced proliferation of AtT20 cells and that JQ1 also significantly increased apoptosis of AtT20 cells. Furthermore, flow cytometry analysis and cellular senescence data showed that JQ1-treated AtT20 cells did not arrest during cell cycle progression, but instead were detected in the sub G1 phase of the cell cycle. Moreover, AtT20 cells did not resume proliferating after removal of JQ1 from the media, indicating that JQ1 also has a major pro-apoptotic effect on AtT20 cells. This is consistent with our data from human NET cell lines, which indicate that JQ1 is more efficacious than PFI-1, and this difference may be due to variations in potency, cell permeability and bioavailability of the two inhibitors, when used at the same concentration (Muller* et al.* 2011, Fish* et al.* 2012, Picaud* et al.* 2013, Lines* et al.* 2017). JQ1 treatment almost completely abolished AtT20 cell proliferation and was therefore more efficacious in the AtT20 cells than in pancreatic NET (BON-1) and broncho-pulmonary NET (H727 and H720) cell lines, as reported in our previous studies (Lines* et al.* 2017). Thieno-diazepines similar to JQ1 are currently undergoing clinical trials for a number of different cancers (Matzuk* et al.* 2012, Benito* et al.* 2017) and therefore may have potential as a novel therapeutic compound for corticotrophinomas, although the specific pathways altered by this pan-BET inhibitor, especially in the pituitary, still remain to be fully elucidated (Andrieu* et al.* 2016).

Our study also demonstrates that JQ1 treatment resulted in a significant dysregulation of apoptosis-associated genes. Of note, one of the major transcriptional targets of BRD4 is *Myc*, and downregulation of *Myc* is considered to be one of the key anti-tumour actions of JQ1 (Delmore* et al.* 2011, Puissant* et al.* 2013, Bandopadhayay* et al.* 2014, Wong* et al.* 2014, Coude* et al.* 2015, Li* et al.* 2016). However, in pancreatic and bronchial NET cells, *Myc* did not appear to be a key target (Lines* et al.* 2017), and in our current study, JQ1 treatment of AtT20 cells did not decrease *Myc* expression but instead was associated with increased *Myc* expression. These findings support the hypothesis that different mechanisms may be responsible for the efficacy of JQ1 in NETs, and these may involve the apoptotic pathway, as indicated by our RNA-Seq analysis that revealed four apoptosis-associated genes (*Capn9*, *Dffb*, *Birc3* and *Nfkb1*) to be downregulated by JQ1 treatment. *Birc3* encodes cIAP2, which is an E3 ubiquitin protein ligase that is a member of the inhibitors of apoptosis proteins (IAP) family, and increases cIAP2 expression that can lead to evasion of caspase-mediated apoptosis in different cancers including gastric and gallbladder cancers and glioblastoma (Wang* et al.* 2016, Jiang* et al.* 2017, Gowda Saralamma* et al.* 2018). In addition, cIAP2 has been reported to activate NFκB (encoded by *Nfkb1*) signalling, which also has roles in apoptosis regulation (Tchoghandjian* et al.* 2013, Jiang* et al.* 2017). Moreover, previous studies have linked BET proteins to the regulation of NFκB signalling, via direct NFκB transcriptional regulation as well as by the binding of Brd4 to acetylated NFκB co-activating proteins (reviewed by Hajmirza* et al.* 2018). These observations therefore suggest that modulation of NFκB signalling may be an important mechanism of action of JQ1 in NETs.

Our study also revealed that the only proliferation-associated pathway significantly altered by JQ1 treatment of AtT20 cells was the anti-proliferative SSTR2 pathway. This indicates that the predominant action of JQ1 is likely through apoptosis; however, somatostatin analogues that target somatostatin receptors are effective in the treatment of somatotrophinomas, a pituitary NET, although their efficacy in treating corticotrophinomas is limited (Feelders* et al.* 2018). There are five somatostatin receptors, SSTR1–5, and SSTR2 and 5 are predominantly involved in hormone secretion, whilst SSTR1, 2, 3, 4 and 5 have roles in apoptosis and proliferation (Grozinsky-Glasberg* et al.* 2008). Pasireotide that has high affinity for SSTR1, 2, 3 and 5 has been reported to decrease cortisol levels in patients with Cushing’s disease, although this was associated with hyperglycaemia-related adverse events in approximately 75% of patients (Colao* et al.* 2012). In contrast, octreotide, which predominantly targets SSTR2 that is one of the most frequently expressed SSTRs in pituitary adenomas, has been reported to have no effect on ACTH secretion in patients with Cushing’s disease or proliferation of AtT20 cells. This is likely because SSTR5 is the most abundant SSTR in corticotrophinomas; however, both AtT20 cells and human corticotrophinomas have been show to express SSTR2, 3 and 5 (Murasawa* et al.* 2014, Behling* et al.* 2018). Thus, our results suggest that using a somatostatin analogue that targets multiple SSTR’s, for example, paseriotide, may be preferable to octreotide, that predominantly targets SSTR2. In addition, our results, which demonstrate that JQ1 treatment can significantly decrease AtT20 cell proliferation and downregulate expression of SSTR2, appear to be paradoxical because SSTR2 is reported to exert anti-proliferative actions (Panetta & Patel 1995, Grozinsky-Glasberg* et al.* 2008). A possible explanation for this apparent paradox may be provided by the reported formation of heterodimers by SSTRs (Duran-Prado* et al.* 2008). For example, SSTR3 is reported to form heterodimers with SSTR2, and as SSTR3 is pro-apoptotic, the observed downregulation of SSTR2 by JQ1 could reduce SSTR2-SSTR3 heterodimer formation but enhance SSTR3-SSTR3 homodimer formation that will increase SSTR3-induced apoptosis. (Theodoropoulou & Stalla 2013). Hence, JQ1 treatment could provide a novel medical approach for modulation of apoptosis-associated somatostatin signalling in patients with corticotrophinomas.

In addition to increasing apoptosis, our results showing that JQ1 can reduce the hypersecretion of ACTH may also provide a possible treatment for Cushing’s syndrome, which is one of the key morbidities of corticotrophinomas (Ntali* et al.* 2013). Thus, JQ1 treatment of AtT20 cells that have been reported to actively secrete ACTH (Buonassisi* et al.* 1962, Gong* et al.* 2014, Asari* et al.* 2017) significantly decreased levels of ACTH in cell media by 48 h and this was preceded by a significant reduction in the expression of *Pomc*, which encodes pre-pro-opiomelanocortin (pre-pro-POMC) that is cleaved to give rise to multiple peptide hormones, including ACTH (Rousseau* et al.* 2007). Therefore, JQ1 may downregulate ACTH secretion by suppressing expression of the protein it is cleaved from in a BET-mediated mechanism. In addition, our results showed a significant decrease in POMC expression after 24 h JQ1 treatment, but secretion of ACTH was not significantly reduced until 48 h after treatment. This is likely because of the time required for the cells to process the POMC protein into ACTH and the fact that the data collected was cumulative, with total ACTH in cell media measured at each time point. Therefore these observations suggest that JQ1 may have a potential to decrease the growth of corticotrophinomas, as well as reduce their ability to secrete ACTH. However, it is important to note that all these studies were performed in an *in vitro* cell line model, and hence these findings require further confirmation by *in vivo* or *ex vivo* investigations before considering a direct clinical utility for treating JQ1 in corticotrophinomas.

Overall, our studies indicate that JQ1 can significantly decrease proliferation and increase apoptosis of the ACTH-secreting (corticotrophinoma) cell line, AtT20, as well as reduce its ACTH secretion. Thus, BET inhibition may provide a novel therapeutic approach for corticotrophinomas possibly through transcriptional regulation of genes including NFκB, SSTR2 and POMC.

## Supplementary Material

Supplementary Figure 1

Supplementary Figure 2

Supplementary Figure 3

Supplementary Figure 4

Supplementary Figure 5

## Declaration of interest

The authors declare that there is no conflict of interest that could be perceived as prejudicing the impartiality of the research reported.

## Funding

This work was funded by the United Kingdom Medical Research Council (MRC) program Grant G1000467 (K E L, M S and R V T) and R V T is a Wellcome Trust Senior Investigator. P F, S K, S M and C B are supported by the SGC, a registered charity (number 1097737) that receives funds from AbbVie, Bayer Pharma AG, Boehringer Ingelheim, Canada Foundation for Innovation, Eshelman Institute for Innovation, Genome Canada through Ontario Genomics Institute, Innovative Medicines Initiative (EU/EFPIA) (ULTRA-DD grant no. 115766), Janssen, Merck & Co., Novartis Pharma AG, Ontario Ministry of Economic Development and Innovation, Pfizer, São Paulo Research Foundation-FAPESP, Takeda and the Wellcome Trust. H L, B W and D B are supported by a Wellcome Trust Core award to the Wellcome Trust Centre for Human Genetics (090532/Z/09/Z).
