# Supplementary material for: Effects of epigenetic pathway inhibitors on corticotroph tumour AtT20 cells
Source: Endocr Relat Cancer. 2020 Jan 13;27(3):163–74. doi: 10.1530/ERC-19-0448 (PMC7040567; doi:10.1530/ERC-19-0448)
Supplement: Supplementary Figure 1 [file supplementary_figure_1.pdf]

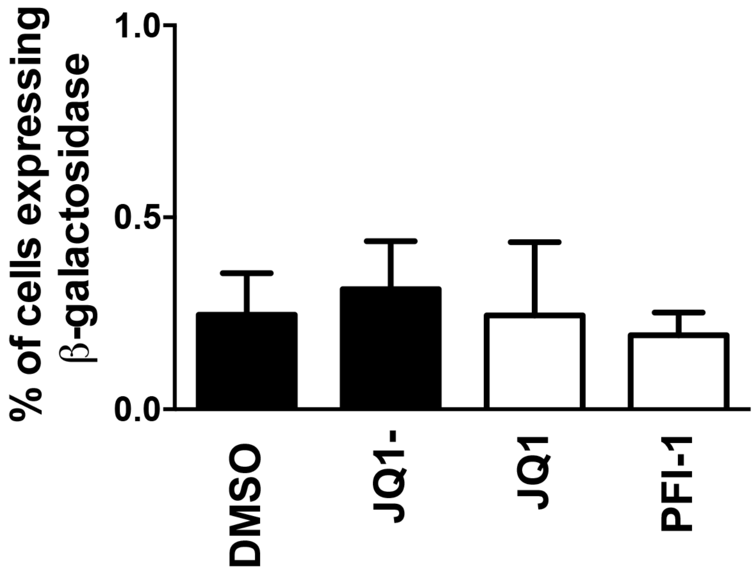

Supplementary Figure 1. Cellular senescence analysis. The level of cellular senescence of AtT20 cells was measured, 96h after compound treatment by determining the percentage of cells staining positively for β-glalactosidase.
