# Supplementary material for: Effects of epigenetic pathway inhibitors on corticotroph tumour AtT20 cells
Source: Endocr Relat Cancer. 2020 Jan 13;27(3):163–74. doi: 10.1530/ERC-19-0448 (PMC7040567; doi:10.1530/ERC-19-0448)
Supplement: Supplementary Figure 2 [file supplementary_figure_2.pdf]

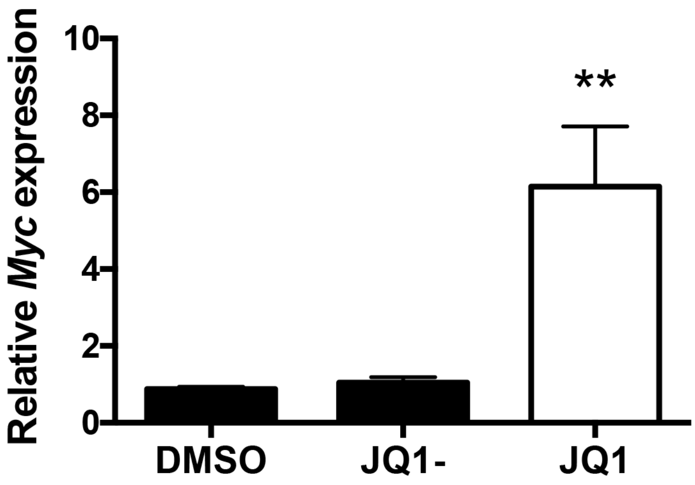

Supplementary Figure 2. Expression of *Myc* in AtT20 cells after JQ1 treatment. Myc expression was evaluated using qRT-PCR after 96h treatment with DMSO and JQ1- control treatments, or JQ1 drug. N=4 biological replicates; **$p<0.005$ by one-way annova relative to DMSO control.
