# Supplementary material for: Effects of epigenetic pathway inhibitors on corticotroph tumour AtT20 cells
Source: Endocr Relat Cancer. 2020 Jan 13;27(3):163–74. doi: 10.1530/ERC-19-0448 (PMC7040567; doi:10.1530/ERC-19-0448)
Supplement: Supplementary Figure 4 [file supplementary_figure_4.pdf]

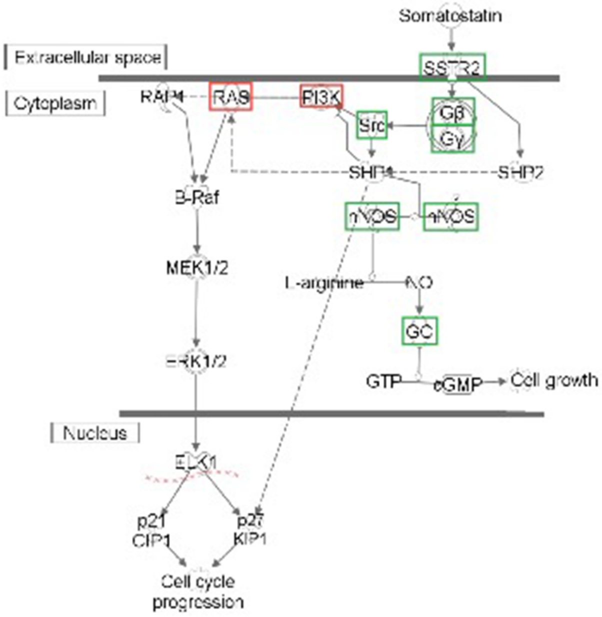

Supplementary Figure 4. Ingenuity pathway analysis indicating somatostatin receptor 2 proliferation-pathway components significantly altered after JQ1 treatment. Genes significantly up-regulated in JQ1 versus JQ1- AtT20 cells are indicated in red boxes and genes significantly down-regulated in JQ1 versus JQ1- AtT20 cells are indicated in green boxes; fold changes and p values are provided in Table 2.
