# Supplementary material for: Effects of epigenetic pathway inhibitors on corticotroph tumour AtT20 cells
Source: Endocr Relat Cancer. 2020 Jan 13;27(3):163–74. doi: 10.1530/ERC-19-0448 (PMC7040567; doi:10.1530/ERC-19-0448)
Supplement: Supplementary Figure 5 [file supplementary_figure_5.pdf]

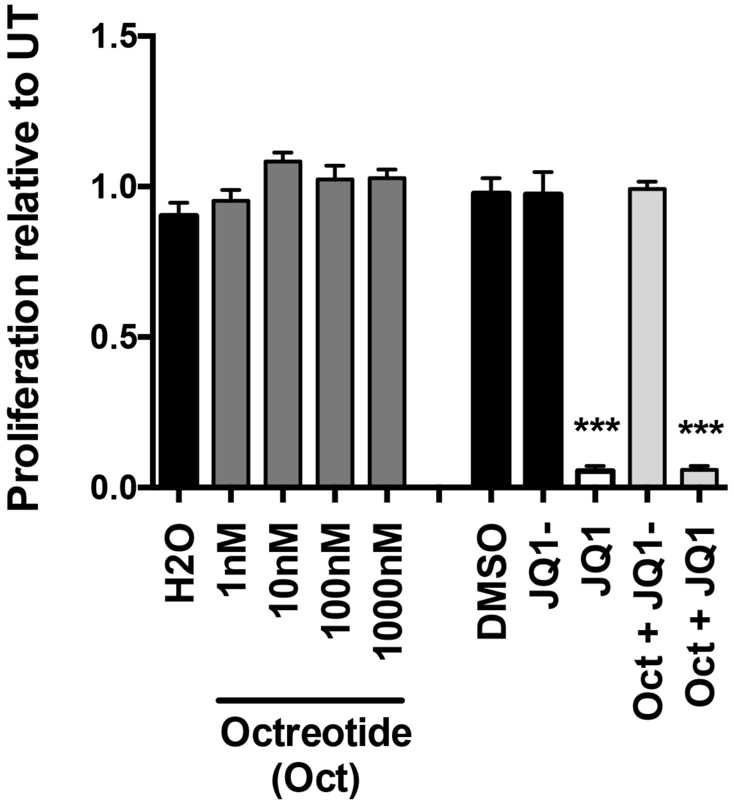

Supplementary Figure 5. Octreotide and JQ1 combination treatment of AtT20 cells. AtT20 cells were treated with increasing doses of Octreotide for 96h and a combination of 1000nM Ocreotide with 1µM JQ1. Data is represented relative to untreated (UT) cells. DMSO, JQ1- and H20 were all used as negative controls. ***p<0.0005.
